# Comparing the first and second waves of COVID-19 in a tertiary university hospital in Barcelona

**DOI:** 10.12688/f1000research.73988.1

**Published:** 2021-11-24

**Authors:** Yolima Cossio, Marta-Beatriz Aller, Maria José Abadias, Jose-Manuel Domínguez, Maria-Soledad Romea, Maria-Àngels Barba, Maria-Isabel Rodríguez, Antonio Roman, Albert Salazar

**Affiliations:** 1 Hospital Universitari Vall d’Hebron, Barcelona, Spain; 2 Health Services Research Group, Institut de Recerca Vall d’Hebron, Barcelona, Spain

**Keywords:** covid-19, hospital management, tertiary hospital

## Abstract

Background:

Hospitals have constituted the limiting resource of the healthcare systems for the management of the COVID-19 pandemic. As the pandemic progressed, knowledge of the disease improved, and healthcare systems were expected to be more adapted to provide a more efficient response. The objective of this research was to compare the flow of COVID-19 patients in emergency rooms and hospital wards, between the pandemic's first and second waves at the University Hospital of Vall d’Hebron (Barcelona, Spain), and to compare the profiles, severity and mortality of COVID-19 patients between the two waves.

Methods:

A retrospective observational analysis of COVID-19 patients attending the hospital from February 24 to April 26, 2020 (first wave) and from July 24, 2020, to May 18, 2021 (second wave) was carried out. We analysed the data of the electronic medical records on patient demographics, comorbidity, severity, and mortality.

Results:

The daily number of COVID-19 patients entering the emergency rooms (ER) dropped by 65% during the second wave compared to the first wave. During the second wave, patients entering the ER were significantly younger (61 against 63 years old p<0.001) and less severely affected (39% against 48% with a triage level of resuscitation or emergency; p<0.001). ER mortality declined during the second wave (1% against 2%; p<0.000). The daily number of hospitalised COVID-19 patients dropped by 75% during the second wave. Those hospitalised during the second wave were more severely affected (20% against 10%; p<0.001) and were referred to the intensive care unit (ICU) more frequently (21% against 15%; p<0.001). Inpatient mortality showed no significant difference between the two waves.

Conclusions:

Changes in the flow, severity and mortality of COVID-19 patients entering this tertiary hospital during the two waves may reflect a better adaptation of the health care system and the improvement of knowledge on the disease.

## Introduction

The COVID-19 pandemic has challenged healthcare systems around the world. ^1^ Hospitals with the capacity to undertake critical patients in intensive care units (ICUs) have constituted the limiting resource of the healthcare systems for the management of the pandemic. ICUs possess essential equipment such as mechanical ventilators and monitoring devices that have enabled the provision of vital support to COVID-19 patients developing a severe form of the disease. ^2^ As a consequence, hospitals, especially tertiary ones, have had to reorganize their activity to make available the largest number of beds for treating COVID-19 patients, including ICU beds, to manage the progressive surge in demand, while trying to maintain essential hospital services. ^3^

As many other countries, Spain has been seriously affected by the COVID-19 pandemic, and a significant number of confirmed cases and deaths have been reported. The first case of COVID-19 was confirmed on January 31 in La Gomera in the Canary Islands. Six weeks later, on March 14, Spain declared a state of emergency and imposed rigorous lockdown measures to the population, ^4^ that enabled a sustained decrease in the accumulated incidence of COVID-19 in the population. ^5^^,^^6^ After the end of the first pandemic wave, the measures to contain the disease were relaxed during the summer, and the incidence of cases began to rise slowly in some Spanish regions such as Catalonia, starting the second wave.

Although we do not have conclusive data about a potential increase of aggressiveness of the virus, both the virus and our knowledge of the disease have evolved over time, as well as the adaptation of health systems to cope with the pandemic. We could therefore expect differences in the number, severity and outcomes of patients admitted in healthcare services, including tertiary hospitals. Several studies pointed out differences in the epidemiological and clinical behaviour of the pandemic waves in terms of severity, transmission and dissemination of the virus. ^7^^,^^8^ Incidence levels showed different patterns between waves, with the number of cases being higher in the second wave in comparison to the first one, probably as a consequence of the shortage of diagnostic testing in the first wave, causing declared numbers underestimating real numbers. ^8^ Moreover, the second wave showed a behaviour of multiple peaks that may reflect the mobility of the population, ^9^ while the incidence decrease may be connected to an increase in containment measures. ^9^^,^^10^ There have been significant improvements in the clinical knowledge of the disease that may have affected the flow of hospitalized patients, as well as their severity and outcome. These include a better approach to treatment, ^11^^,^^12^ and a better understanding of prognostic factors ^13^ that may have served to optimize treatment, and resource management strategies in the care of COVID-19 patients. Finally, the adaptation of healthcare systems may have also modified the profile and flow of patients entering the hospitals. In Catalonia, there has been a progressive adaptation of health services to better allocate patients according to the complexity of care needed. ^14^ While in the first wave COVID-19 care was mainly hospital-centered, with scarce territorial coordination, there has been a progressive increase in coordination between healthcare levels, including the definition of clear roles and care protocols for the primary care. ^15^ In addition, key strategies have been implemented to protect vulnerable groups, such as the systematic COVID-19 screening in nursing homes ^16^ and vaccination campaigns. ^17^

Tertiary hospitals are expected to treat complex patients requiring a more specialized care. During the first wave, hospitals have been the first contact with the healthcare system for many COVID-19 patients, almost resulting in its collapse. As the pandemic progressed, knowledge of the disease improved and healthcare systems adapted to provide a more efficient response. As a consequence, tertiary hospitals might have mainly received the most severe COVID-19 cases. However, few data are available to analyse how the profile and severity of COVID-19 patients entering tertiary hospitals has evolved over time. The aim of this study was to compare the flow, profile, severity and mortality of COVID-19 patients, during the first and second waves of the pandemic at the University Hospital of Vall d’Hebron (Barcelona).

## Methods

### Ethical considerations and consent

This study was approved by the Clinical Research Ethics Committee at the Hospital Universitari Vall d’Hebron from Barcelona (Spain), in session Number 497 on 30 July 2021. No consent from the participants was required for this study since this is a retrospective research study based on anonymous and de-identified data.

### Study design and setting

This retrospective, exploratory, observational analysis compared demographic, clinical and outcome data of COVID-19 patients attending the Vall d’Hebron University Hospital (HUVH), from February 24 to April 26, 2020 (first wave) and from July 24, 2020 to May 18, 2021 (second wave). The HUVH is a 1,100-bed university hospital in Barcelona, the second largest hospital in Spain, and is part of the Catalan Public Healthcare System, which provides universal coverage to the population. The hospital works in close coordination with other healthcare organisations in the region, including one secondary care hospital, three intermediate care centres and 19 primary care centres. The HUVH hosts over 7,500 healthcare professionals and covers a population of about 450,000 people.

**Figure 1.**
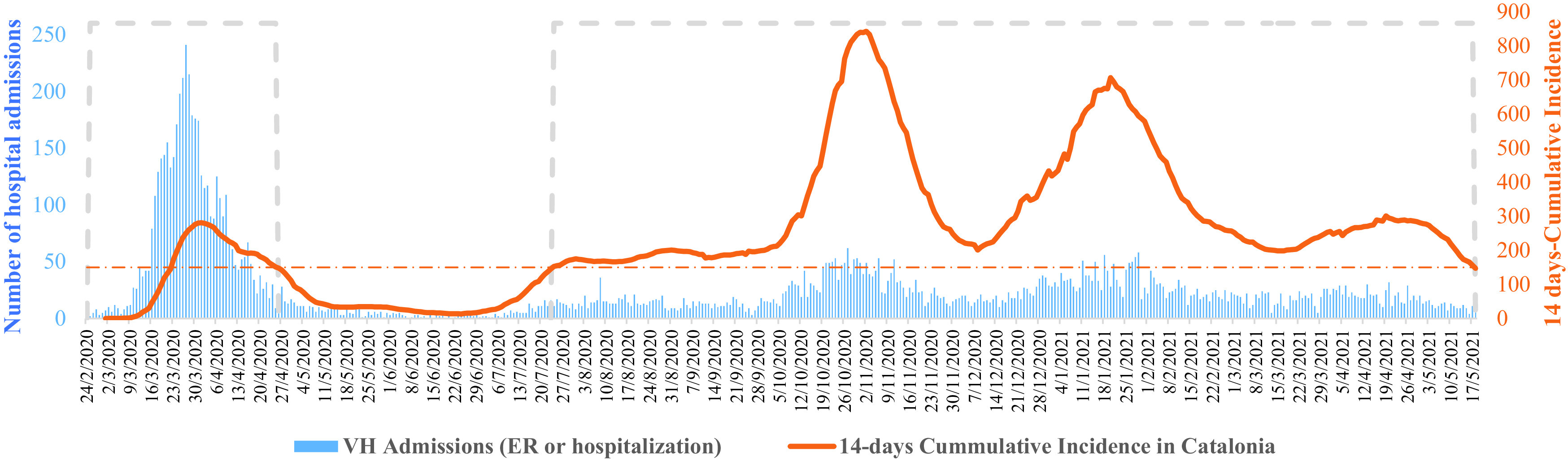
14 days-CI in Catalonia and COVID-19 hospital admissions (emergency or hospitalization). The orange dashed line shows the cumulative incidence threshold that has been defined as a very high-risk situation in Spain. The blue dashed line represents the two waves of the pandemic in Catalonia according to the criteria defined in the article.

Waves have been defined based on the 14-day cumulative incidence (CI) in Catalonia, which is published by the Catalan Health Department, and is defined as the total number of confirmed cases in the prior 14 days/100,000 population. A CI value equal to or lower than 150 has been set to define the boundaries of the waves, as this value was considered the threshold for a high-risk situation in Spain according to the health authorities. ^19^ We defined February 24, 2020 as the start date of the first wave, when the first case of COVID-19 was reported in Catalonia ( Figure 1). The peak of CI during the first wave was on April 1, with a value of 281.26, and the end of the first wave was on April 26, when the CI fell below 150 (147.85). The second wave started on July 24, when the CI exceeded 150 (153.4), reached its peak on October 31 (840.27), and ended on May 18, when the CI fell below 150 (146.89). Data was extracted and frozen on August 16, 2021.

### Participants

The study included 8,684 distinct COVID-19 patients who attended the emergency rooms (ER) and/or were hospitalised. Cases were identified using the ICD-10-CM diagnostic codes recorded in the hospital-discharge data: B34.2 and B97.29 (from February 2020) and ICD-10 code U07.1 (from July 1, 2020). Discharge diagnoses of hospitalised patients were registered by specialised coders. To guarantee that we were analysing and comparing data from patients affected by the COVID-19 disease, we only included: i) patients whose primary diagnosis was COVID-19 or, ii) patients with a secondary COVID-19 diagnosis following a first diagnosis of a respiratory system disease (ICD-10-CM code starting by J), or who were described with a Diagnosis Related Groups (DRG) 137 (Infections in major lung inflammations) or 139 (other pneumonia). The selection criteria for the patients attending the ER was to have a confirmed COVID-19 diagnosis, regardless of whether it was a primary or secondary diagnosis.

### Variables and data sources

To compare the inpatient and emergency flow activity of the HUVH between waves, we created an indicator measuring the total daily number of patients entering the hospital. To compare the characteristics of COVID-19 patients who entered the hospital emergency department and/or were hospitalized, we used data on patient demographics (sex and age) and comorbidity (the adjusted morbidity groups [GMA]). GMA is a validated morbidity measurement developed and adapted to the Spanish Healthcare System that classifies the population into seven morbidity groups, taking into account the typology of their diseases (acute, chronic, or oncological), and in the case of chronic disease, identifying whether it is a single or a multimorbidity. Two additional groups refer to pregnant/childbirth women and populations without previous pathologies. ^20^^,^^21^

To compare the severity of patients attending the ER, we included the type of emergency as defined by the structured triage system for emergency services implemented in Catalonia, which is based on the Australian-Canadian triage systems, and divides the emergencies into five groups: resuscitation, emergent, urgent, less urgent and non-urgent. ^22^ The severity of hospitalised patients’ state was analysed according to the following variables: first, the severity of the episode, as measured by the weight of the diagnosis-related groups (APR- DRG, version 36) and its severity. Changes in the DRG weights of COVID-19-related diagnosis occurred in July 2020, due to 1) the use of the U07.1 diagnosis code for COVID-19, 2) the number of patients accessing the ICU and 3) the overall length of stay (LOS) during the hospitalization and the ICU-specific LOS. Finally, to compare the mortality rates between the two waves, we included three variables: overall mortality during the hospitalization, three-day mortality and mortality of patients who entered the ICU.

Data was obtained from the electronic medical records and extracted using structured query language (SQL), to create and anonymized database. The data collection period was defined as per the hospital admission date and only discharged patients’ data was included.

### Data analysis

Continuous variables were reported as mean and standard deviation (SD), and median and interquartile range (IQR). Categorical variables were presented as frequency rates and percentages. Continuous variables were compared using the Mann-Whitney U-test, while comparisons of categorical variables were performed using Chi square tests. All analyses were performed using STATA 13.0 (STATA Corp.). A p-value lower than 0.05 was considered as statistically significant.

## Results

### Emergency care

**Figure 2.**
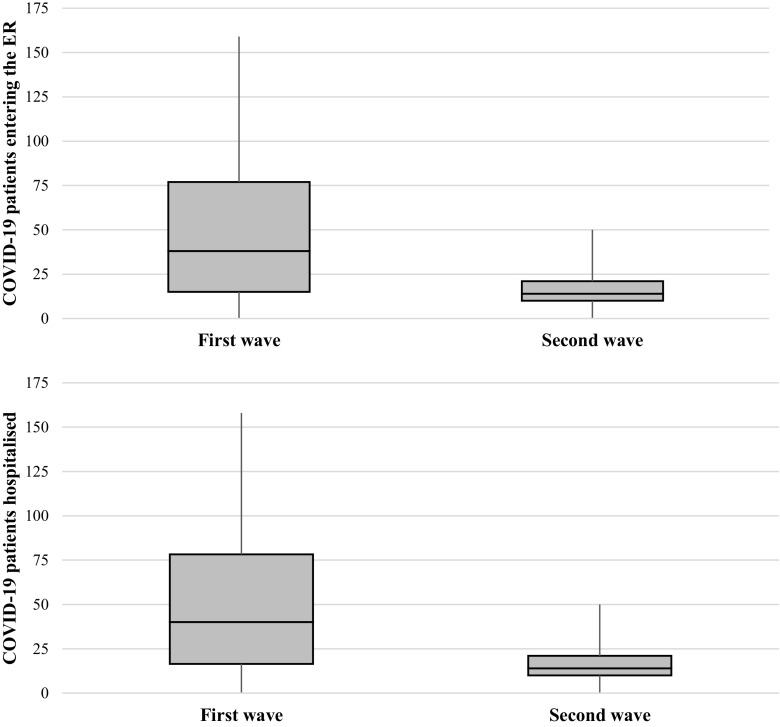
Distribution of the number of hospital admissions per day during the first and second waves.

A 65% drop in the daily number of COVID-19 patients entering the ER was observed during the second wave compared to the first wave (17.5 against 49.8 patients per day on average, respectively). The distribution of ER admissions is shown in Figure 2. During the second wave, patients entering the ER were significantly younger (median age of 61 against 63) ( Table 1). No significant difference was observed in the percentage of women entering the ER (47.0% and 47.6% during the second and first waves, respectively). During the second wave, patients entering the ER were less severely affected (39.1% against 48.5%, with a triage level of resuscitation or emergency), and the ER mortality significantly declined from 2.3% to 1.2%.

**Table 1.** Characteristics, type of emergency and mortality of COVID-19 patients entering the hospital. IQR = interquartile range; GMA = adjusted mortality group. Bold p-values represent statistically significant values.

	First wave (Feb 24, 2020 - Apr 26, 2020)	Second wave (Jul 24, 2020 - May 18, 2021)	p-value
**n episodes**	3037	5043	
**Demographic characteristics of patients**			
***Sex***			
Women (n (%))	1447 (47.6%)	2375 (47.0%)	0.631
***Age***			
Mean (DE)	62.4 (19.4)	58.4 (22.7)	
Median (IQR)	63 (29)	61 (34)	**0.000**
< 18 years (n (%))	48 (1.58%)	272 (5.39%)	**0.000**
18-35 years (n (%))	209 (6.88%)	512 (10.1%)	**0.000**
35-49 years (n (%))	543 (17.8%)	963 (19.0%)	0.174
50-64 years (n (%))	789 (25.9%)	1137 (22.5%)	**0.000**
65-79 years (n (%))	779 (25.6%)	1127 (22.3%)	**0.001**
> 80 years (n (%))	669 (22.0%)	1032 (20.4%)	0.095
**Morbidity**			
***Morbidity groups (GMA)***	*2992*	*4967*	
No previous pathologies	137 (4.57%)	235 (4.73%)	0.757
Acute pathologies	50 (1.67%)	257 (5.17%)	**0.000**
Pregnancy and childbirth	17 (0.56%)	30 (0.60%)	0.841
Chronic pathologies in 1 system	237 (7.92%)	449 (9.03%)	0.086
Chronic pathologies in 2 or 3 systems	672 (22.4%)	1025 (20.6%)	0.054
Chronic pathologies in > 3 systems	1638 (54.7%)	2567 (51.6%)	**0.008**
Active neoplasm	124 (4.14%)	190 (3.82%)	0.477
**Type of emergency**	*2992*	*4967*	
Resuscitation + emergency (n (%))	1453 (48.5%)	1944 (39.1%)	**0.000**
Urgency (n (%))	1307 (43.6%)	2643 (53.2%)	
Minor urgency + non urgent (n (%))	232 (7.75%)	380 (7.65%)	
**Mortality**			
Mortality (n(%))	70 (2.30%)	61 (1.20%)	**0.000**

### Inpatient care

A 75% drop in daily hospitalised COVID-19 patients was observed during the second wave compared to the first wave (7.9 against 32.0 mean patients per day, respectively). The distribution of hospital admissions is shown in Figure 2. No significant differences were found when comparing the median age ( Table 2); however, patients older than 80 represented a lower percentage among hospitalised patients during the second wave in comparison to the first wave (8.2% against 11.6%), while patients younger than 18 represented a higher percentage during the second wave (2.7% against 1.7%). The percentage of hospitalized women was significantly lower during the second compared to the first wave (38.8% against 43.9%). In terms of basal morbidity, the most significant difference between waves was found in the population with acute pathologies, which increased during the second wave (5.6% against 2.1%).

**Table 2.** Characteristics, severity and mortality of COVID-19 hospitalised patients. IQR = interquartile range. Bold p-values represent statistically significant values.

	First wave (February 24, 2020 - April 26, 2020)	Second wave (July 24, 2020 - May 18, 2021)	p-value
n episodes	2013	2367	
**Demographic characteristics of patients**			
***Sex***			
Women (n (%))	885 (43.9%)	920 (38.8%)	**0.001**
***Age***			
Mean (DE)	59.6 (17.3)	58.7 (17.1)	
Median (IQR)	60 (23)	61 (22)	0.296
< 18 years (n (%))	35 (1.7%)	63 (2.7%)	**0.040**
18-35 years (n (%))	115 (5.7%)	129 (5.4%)	0.705
35-49 years (n (%))	389 (19.3%)	471 (19.8%)	0.634
50-64 years (n (%))	676 (33.5%)	778 (32.8%)	0.617
65-79 years (n (%))	564 (28.0%)	732 (30.9%)	0.036
> 80 years (n (%))	234 (11.6%)	194 (8.2%)	**0.000**
**Morbidity**			
***Morbidity groups (GMA)***	*1895*	*2245*	
No previous pathologies (n (%))	94 (4.96%)	87 (3.87%)	0.099
Acute pathologies (n (%))	40 (2.11%)	125 (5.56%)	**0.000**
Pregnancy and childbirth (n (%))	11 (0.58%)	4 (0.17%)	**0.033**
Chronic pathologies in 1 system (n (%))	175 (9.23%)	208 (9.26%)	0.913
Chronic pathologies in 2 or 3 systems (n (%))	503 (26.5%)	534 (23.7%)	0.060
Chronic pathologies in > 3 systems (n (%))	992 (52.3%)	1184 (52.7%)	0.625
Active neoplasm (n (%))	80 (4.22%)	103 (4.58%)	0.534
**Severity of the episode**	*2018*	*1847*	
Severity, weigh (DRG) (mean (DE))	1.1 (1.8)	1.4 (1.9)	
Severity, weight (DRG) (median (IQ))	0.4 (0.2)	0.7 (0.2)	**0.000**
Higher degree of severity (n (%))	202 (10.0%)	479 (20.2%)	**0.000**
**ICU care**			
n episodes (n (%))	309 (15.3%)	488 (20.6%)	**0.000**
**Lenght of stay**			
Lenght of stay hospitalization (mean (DE))	10.1 (13.7)	11.3 (14.5)	
Lenght of stay hospitalization (median (IQR))	5.9 (5.8)	6.6 (8.9)	**0.007**
Lenght of stay ICU (mean (DE))	19.3 (17.3)	16.6 (17.1)	
Lenght of stay ICU (median (IQR))	15.2 (18.5)	9.8 (19.1)	**0.003**
**Global mortality**			
Mortality (n (%))	199 (9.88%)	221 (9.33%)	0.539
3-days mortality (n (%))	63 (20.3%)	48 (9.8%)	**0.021**
**Mortality of ICU admitted patients**			
Mortality (n (%))	61 (19.7%)	64 (13.1%)	0.518

The median severity of the hospitalizations significantly increased during the second wave, with those patients hospitalised during the second wave being more severely affected (20.2% had the highest DGR severity score compared to 10.0% during the first wave) and were admitted more frequently to the ICU (20.6% against 15.3%). The median length of stay significantly increased during the second wave (6.6 against 5.9 days during the first wave) while the median ICU length of stay was significantly lower during the second wave (9.8 against 15.2 days during the first wave). Finally, there were no significant differences in inpatient nor in ICU mortality between waves, although the three-day-mortality rate was significantly lower during the second wave (2.1% against 3.1% during the first wave).

## Discussion

The study shows differences in the flow and characteristics of hospitalized patients between the first and second waves of the COVID-19 pandemic at the HUVH. While the daily flow of patients entering the hospital during the second wave was lower, hospitalized patients were more severely affected, and a higher proportion were admitted to the ICU. However, during the second wave, we did not observe any increase of the overall mortality among hospitalized patients, despite the increase in severity.

The lower daily flow of COVID-19 patients entering the hospital during the second wave contrasts with the increased incidence officially reported in the population during this period. ^18^ This result reflects the adaptation of the health system to tackle the pandemic, with a greater involvement of other healthcare levels in the diagnosis and management of COVID-19 patients. ^14^ During the first wave, hospitals were challenged with the need to simultaneously provide healthcare to a high number of COVID-19 patients, with hospitals centralizing most of the healthcare activity, and reaching a situation close to the collapse. However, the second wave benefited from a more experienced management and the redistribution of the responsibilities at various levels, stratifying patients and referring them to hospitals only upon need. ^15^^,^^23^ In addition, during the first wave, there was a limited availability of real-time diagnostic tests in primary care, and the diagnosis of COVID-19 was mainly conducted in hospitals, especially on symptomatic patients. ^24^ Conversely, during the second wave, population screenings were conducted and a rapid diagnosis of COVID-19 was performed mainly at the primary care level. These adaptations of the healthcare system during the second wave may have led to a lower daily flow of patients requiring hospitalization.

The severity of COVID-19 patients entering the ER and getting hospitalized differed between waves. COVID-19 patients entering the ER during the second wave were less severely ill, whilst the opposite was observed in hospitalizations. The lack of clinical knowledge on the disease and the messages communicated to the population about hospital collapse during the first wave may have contributed to these differences, as well as significant improvements in the coordination of health services produced during the second wave. Regarding the severity of cases in the ER, we measured this parameter according to the structured triage system implemented in Catalonia. ^22^ It is therefore possible that during the first wave, the lack of knowledge of the disease by the personnel responsible for ER triage led to registering patients with a higher level of urgency. During the first wave the population was encouraged to stay at home. This, added to the population’s fear of infection, may have resulted in people being more likely to seek emergency care only when there was a more severe presentation of the disease, with almost no alternatives to seeking care outside of hospitals. Regarding the severity of hospitalised patients, other factors may be contributing to the observed differences, probably related to the adaptation of the healthcare system. In fact, the higher percentage of COVID-19 patients requiring ICU care and being categorised with the highest degree of severity at discharge, may be reflecting the participation of other healthcare levels in the management of COVID-19 that was observed during the second wave ^14^^,^^23^; this may have resulted in a better stratification of the patients referred to the hospital, depending on the severity of their symptoms. An increased knowledge on prognostic factors may have also affected the severity of hospitalised patients. While during the first wave any patient with a radiologically-diagnosed pneumonia was hospitalised, during the second wave the hospital implemented protocols to hospitalize patients with a COVID-19 diagnostic according to their prognostic factors. Finally, the observed differences are not expected to be caused by differences in the virus, as new strains of the SARS-CoV-2 virus were detected late in Spain. ^25^

In addition to differences in the severity of patients entering the hospital between waves, we observed differences in these patients’ profiles in terms of age, sex and comorbidities. First, younger patients were admitted into the ER during the second wave, which is consistent with the lower severity reported in this period. In addition, during the second wave a lower percentage of COVID-19 patients older than 80 and patients without any described comorbidity were hospitalised. The large number of older patients coming from nursing homes during the first wave, most of which presenting a severe condition, together with actions to improve the protection of this population after the first wave ^26^ and their prompt vaccination around mid-January 2021, ^27^ could explain the differences in age distribution between the two waves. Finally, a lower percentage of women were hospitalized during the second wave compared to the first one. As previously described, being male correlates with a more severe COVID-19 profile. ^28^ The increased severity in the hospitalized patients observed during the second wave is correlated with the lower percentage of hospitalized women.

Finally, we did not observe any increase of the overall mortality during the second wave, despite the greater severity of hospitalised patients. Significant improvements in the clinical knowledge of the disease may contribute to explain this result. A large number of drugs have been tested from the start of the pandemic around the world in order to identify effective therapeutic alternatives. ^29^ This led to the identification of a few safe and effective therapeutic approaches in severe cases of the disease, including anticoagulant therapy and corticosteroids. ^11^^,^^12^

This study has some limitations **,** as it used a limited number of parameters for the analysis. Further analysis including the type of treatments received during the hospitalization, or more detailed information on previous conditions would help gain a better understanding of the differences in the profiles of COVID-19 patients admitted between the pandemic phases, as well as differences in hospital management strategies.

## Conclusions

This comparative study shows a less intensive activity in the second wave of the COVID-19 pandemic in both the ER frequentation and hospitalizations, but more severe profiles of patients receiving inpatient care compared to the first wave. Although severity in hospitalised patients increased over time, no significant differences were observed in the overall mortality rate during the hospitalization. The results may be reflecting a better adaptation of the overall healthcare system, including a better adaptation and coordination of the healthcare services, better understanding of prognostic factors and treatment, and the success of the different strategies to protect more vulnerable populations, including vaccination and protection of the elderly in nursing homes. These results may be helpful to improve current knowledge on the evolution of the pandemics in a tertiary hospital, the most limiting resource in the healthcare system, and may shed light on the adaptation processes of the healthcare systems and their results for incoming waves.

## Data availability

### Underlying data

DRYAD: Comparing the first and second waves of COVID-19 in a tertiary university hospital in Barcelona, https://doi.org/10.5061/dryad.0k6djhb1d. ^30^

This project contains the following underlying data:

- Files_manuscript_COVID12Waves_20210910_.xlsx, that includes: 1) table describing the number of hospital admissions by day and the 14 days accumulated incidence in Catalonia; 2) table describing the profile of patients accessing emergency care, the type of emergency and mortality; and 3) table with data describing the profile of hospitalised patients, and details on their length of stay, UCI stay, severity, and mortality.
- Readme_COVID12Waves.xlsx (a detailed description of variables included in the files).

Data are available under the terms of the Creative Commons Zero “No rights reserved” data waiver (CC0 1.0 Public domain dedication).
