# Supplementary material for: Comparing the first and second waves of COVID-19 in a tertiary university hospital in Barcelona
Source: F1000Res. 2021 Nov 24;10:1197. [Version 1] doi: 10.12688/f1000research.73988.1 (PMC9350435; doi:10.12688/f1000research.73988.1)
Supplement: Peer review history

# Peer review: Reviewer response for version 1

**DOI:** 10.5256/f1000research.77690.r143966

**Published:** 2022-08-03

**Authors:** Andri Frediansyah

**Affiliations:** 1 National Research and Innovation Agency (BRIN), Jakarta Pusat, Indonesia

Cossio et al. investigated the flow of COVID-19 patients in emergency rooms and hospital wards in Barcelona, Spain, between the first and second waves of the pandemic. Additionally, they compared the severity and mortality profiles of COVID-19 patients from the two waves. The entire manuscript is well-written, and the methodology is well-executed. Therefore, I suggest indexing after the following minor revisions:

1. Please define ICD-10-CM in the introductory paragraphs.
2. Please include some hospital-improved strategies as the hospital also improves its facilities after the first wave.
3. Please include the limitations of the study.
4. Please specify the hospital's COVID-testing procedures.
5. Some of the references listed below may be useful for discussing the results.

Is the work clearly and accurately presented and does it cite the current literature?

Yes

If applicable, is the statistical analysis and its interpretation appropriate?

Yes

Are all the source data underlying the results available to ensure full reproducibility?

Yes

Is the study design appropriate and is the work technically sound?

Yes

Are the conclusions drawn adequately supported by the results?

Yes

Are sufficient details of methods and analysis provided to allow replication by others?

Yes

Reviewer Expertise:

Pharmacology and Microbiology

I confirm that I have read this submission and believe that I have an appropriate level of expertise to confirm that it is of an acceptable scientific standard.

## References

[1] : Comparison of first and second waves of COVID-19 through severity markers in ICU patients of a developing country. *J Community Hosp Intern Med Perspect* .2021;11(5) : 10.1080/20009666.2021.1949793 576-584 10.1080/20009666.2021.1949793 34567444PMC8462838

[2] : Comparing the first and the second waves of COVID-19 in Italy: differences in epidemiological features and CT findings using a semi-quantitative score. *Emerg Radiol* .2021;28(6) : 10.1007/s10140-021-01937-y 1055-1061 10.1007/s10140-021-01937-y 34322767PMC8318634

[3] : First and second waves of coronavirus disease-19: A comparative study in hospitalized patients in Reus, Spain. *PLoS One* .2021;16(3) : 10.1371/journal.pone.0248029 e0248029 10.1371/journal.pone.0248029 33788866PMC8011765

[4] : Epidemiological comparison of the first and second waves of the COVID-19 pandemic in Nigeria, February 2020-April 2021. *BMJ Glob Health* .6(11) : 10.1136/bmjgh-2021-007076 10.1136/bmjgh-2021-007076 34794956PMC8602923

[5] : Comparison of the clinical presentation across two waves of COVID-19: a retrospective cohort study. *BMC Infect Dis* .2022;22(1) : 10.1186/s12879-022-07413-3 423 10.1186/s12879-022-07413-3 35505306PMC9063242

[6] : Main differences between the first and second waves of COVID-19 in Madrid, Spain. *Int J Infect Dis* .2021;105: 10.1016/j.ijid.2021.02.115 374-376 10.1016/j.ijid.2021.02.115 33684560PMC7934652

[7] : Comparison of Two Waves of COVID-19 in Critically Ill Patients: A Retrospective Observational Study. *Int J Nephrol* .2022;2022: 10.1155/2022/3773625 3773625 10.1155/2022/3773625 35665075PMC9161135

# Peer review: Reviewer response for version 1

**DOI:** 10.5256/f1000research.77690.r143967

**Published:** 2022-07-13

**Authors:** Guglielmo Mantica

**Affiliations:** 1 IRCCS Ospedale Policlinico San Martino, Genova, Italy

This is an interesting manuscript about the difference between ED admissions between first and second wave at a hospital in Barcelona, Spain. The abstract and introduction are well written. Methods are well designed and clear. 

 Here some minor concerns:

- *“The study included 8,684 distinct COVID-19 patients who attended the emergency rooms (ER) and/or were hospitalised.”* This is already a result. Please move it to the correct section.
- Please specify in the methods if in your hospital there have been differences/changes in COVID swab tests during the two different waves (type of diagnostic test) since this might lead to differences in the correct identification of patients.
- Please expand and declare all limitations.
- Some authors found differences between admissions during the first and second waves also for Non Covid patients (doi: 10.1016/j.ajem.2020.11.046.). Do you have any data available from your hospital? Otherwise just make a comment in the discussion session.

Is the work clearly and accurately presented and does it cite the current literature?

Yes

If applicable, is the statistical analysis and its interpretation appropriate?

Yes

Are all the source data underlying the results available to ensure full reproducibility?

Yes

Is the study design appropriate and is the work technically sound?

Yes

Are the conclusions drawn adequately supported by the results?

Yes

Are sufficient details of methods and analysis provided to allow replication by others?

Yes

Reviewer Expertise:

Urology

I confirm that I have read this submission and believe that I have an appropriate level of expertise to confirm that it is of an acceptable scientific standard.

## References

[1] : Non-COVID-19 admissions to the emergency department during the pandemic second wave in Italy: What is changed from the first wave?. *The American Journal of Emergency Medicine* .2021;45: 10.1016/j.ajem.2020.11.046 625-626 10.1016/j.ajem.2020.11.046 PMC769029933303298
